# Early-Warning System for Antimicrobial Resistance in *Campylobacter* in the Broiler Production Chain from High-Level Indicators—A Graph-Based Machine Learning and Bayesian Approach

**DOI:** 10.3390/vetsci12111080

**Published:** 2025-11-12

**Authors:** Szilveszter Csorba, Krisztián Vribék, Máté Farkas, Edith Alice Kovács, Dániel Pfeifer, Miklós Süth, Orsolya Strang, Andrea Zentai, Zsuzsa Farkas

**Affiliations:** 1 Department of Digital Food Science, Institute of Food Chain Science, University of Veterinary Medicine, István utca 2., H-1078 Budapest, Hungary; csorba.szilveszter@univet.hu (S.C.); vribek.krisztian@univet.hu (K.V.); farkas.mate@univet.hu (M.F.); strang.orsolya@univet.hu (O.S.); farkas.zsuzsa@univet.hu (Z.F.); 2 National Laboratory of Infectious Animal Diseases, Antimicrobial Resistance, Veterinary Public Health and Food Chain Safety, University of Veterinary Medicine Budapest, István utca 2., H-1078 Budapest, Hungary; kovacsea@math.bme.hu (E.A.K.); suth.miklos@univet.hu (M.S.); 3 Department of Analysis and Operations Research, Budapest University of Technology and Economics, Műegyetem rkp. 3., H-1111 Budapest, Hungary; pfeiferd@math.bme.hu; 4 Institute of Food Chain Science, University of Veterinary Medicine, István utca 2., H-1078 Budapest, Hungary

**Keywords:** AMR, One Health, food safety, *Campylobacter*, broiler chicken, machine learning, indicator, Bayesian analysis

## Abstract

Forecasting antimicrobial resistance (AMR) is critical for public health, yet most models neglect the interconnected nature of agricultural systems. Focusing on ciprofloxacin resistance in *Campylobacter jejuni*—a leading foodborne pathogen in poultry—this study aims to develop a probabilistic framework for identifying high-risk environmental conditions. We employed a graph-based machine learning and Bayesian approach, integrating and discretizing data from international databases. An exploratory classification with XGBoost and SVC was followed by core analysis using a Generalized Naive Bayes (GNB) model for feature selection and a Bayesian Network (BN) to uncover conditional dependencies. The GNB model identified pesticides, land use, and precipitation as key features. The BN revealed a complex web of interactions, showing that resistance probability is highly context-dependent. Precipitation was a critical effect modifier; for example, expanded land use correlated with an 18.3% increase in resistance probability during dry conditions but a 73.7% decrease during wet periods. Scenarios with low and high precipitation were associated with high risk, indicating multiple environmental pathways. Our results demonstrate that Bayesian networks can effectively model the complex, non-linear relationships driving AMR. Ciprofloxacin resistance emerges from system-wide interactions rather than isolated factors. This approach provides a valuable framework for generating hypotheses and supports the development of early-warning systems for targeted antimicrobial stewardship in poultry production.

## Simple Summary

When bacteria become resistant to antibiotics, it makes infections harder to treat. This is a major problem in our food supply, especially with a common foodborne bacterium called *Campylobacter*, which is often found in chickens, and is becoming resistant to a key antibiotic, ciprofloxacin. Our research aimed to predict when this resistance is likely to increase by looking at indirect but easy-to-measure factors like land use, pesticide application, and climatic variables. We used a special type of computer model that can map out how these factors are connected. We found that the risk of antibiotic resistance does not come from a single cause, but from the interaction of many conditions. For example, a certain agricultural activity can mean high risk during dry periods, but low risk when it is very wet. By understanding these complex relationships, our model can help farmers and policymakers take smarter, targeted actions to reduce antibiotic resistance, protecting both animal and human health.

## 1. Introduction

Forecasting systems are playing an increasingly important role in public health and food safety by anticipating emerging risks and supporting timely, evidence-based decisions [1,2]. Data-driven approaches, fueled by large and diverse datasets, are now used to identify patterns and predict future outcomes [3]. In recent years, machine learning (ML) and statistical modeling techniques have significantly enhanced the predictive capacity of these systems, offering improved accuracy, scalability, and adaptability [2,4]. Within the domain of AMR research, predictive models have been applied to forecast resistance trends in both clinical [5] and agricultural contexts [6,7], supporting antimicrobial stewardship and informing policy [8]. However, despite these advances, most existing models often neglect the structured and dynamic nature of real-world systems [9]. To address this gap, data analytical frameworks—such as graph-based machine learning and Bayesian modeling [10]—offer new opportunities.

Among zoonotic pathogens, *Campylobacter jejuni* (*C. jejuni*) remains a leading cause of foodborne illness globally [11,12]. In poultry production, *C. jejuni* is a major zoonotic pathogen [13,14], responsible for the majority of bacterial foodborne illnesses in humans, with 148,181 cases across the EU [11,14], with increasing reports of fluoroquinolone resistance [15,16]. The historical use of antibiotics for growth promotion and prophylaxis in broilers has played a key role in resistance development [17]. Resistant strains can persist throughout the production cycle and contaminate meat products, acting as a reservoir for human infections [18,19]. While interventions such as improved biosecurity, bacteriophage therapy, and vaccination have been proposed to reduce *Campylobacter* colonization in poultry [19,20], fluoroquinolone resistance is still widespread [16,20]. For example, ciprofloxacin resistance in *C. jejuni* ranges from 27.6% to 97.5% in the EU, posing a particular threat [21], given the limited alternative treatments available and the potential for transmission through food and the environment [15].

AMR is a multifaceted One Health issue, driven mainly by antibiotic misuse in both human medicine and animal production [22,23]. However, AMR is also affected by different environmental, agricultural and anthropogenic drivers [24]. Among these, agricultural activities are major contributors to the spread of AMR. For this analysis, key agricultural drivers were selected based on their established role as precursors to AMR or their association with an elevated risk of its emergence, a selection informed by both the literature and expert knowledge [25].

Agricultural activities, particularly the use of fertilizers and pesticides, are major drivers of the spread of AMR. Nitrogen-based fertilizers can alter soil microbiomes and promote the proliferation of antibiotic resistance genes (ARGs) [26,27], while monoculture cropping and intensive land use reduce biodiversity and exacerbate selective pressures [28,29]. Pesticides and herbicides can further co-select for resistance, creating a complex interplay between crop production and AMR [30,31]. Climate change compounds these effects. Rising temperatures accelerate bacterial growth and horizontal gene transfer [32,33], while extreme weather events facilitate the transport of ARGs from agricultural soils to water bodies [34], threatening both environmental and human health [35]. Land use change and intensive farming increase greenhouse gas emissions [36] and also disrupt soil and water ecosystems [37], creating favorable conditions for ARG persistence and transfer [38].

Effective AMR mitigation, therefore, requires not only control measures at the farm level but also predictive surveillance systems that can anticipate resistance trends and support targeted interventions.

Despite growing awareness of the AMR threat in foodborne pathogens, forecasting resistance dynamics in agricultural systems remains scarce. Current surveillance systems are often retrospective in nature, limiting their utility for proactive interventions. Therefore, the primary goal of this study is to create a scenario-based predictive model that functions as an early-warning decision-support tool. The model does not forecast temporal trends but instead identifies high-risk conditions by answering the following question: “What is the probability of observing ciprofloxacin resistance given a specific set of environmental and agricultural indicators?”

## 2. Materials and Methods

### 2.1. Data Description and Preparation

#### 2.1.1. Indicator Data

The indicators we considered relevant were collected from reputable sources, which are authoritative databases providing global statistics on agriculture, food, and the environment. This collection of datasets included a variety of variables relevant to the study, such as agricultural activity, land use, water resources, and other environmental metrics that could potentially influence AMR trends.

In this analysis, the selection of key agricultural drivers was preceded by a structured, multi-step process involving hazard identification, indicator mapping, assessment of available data sources and databases, and subsequent data harmonization. The initial conceptual framework was informed by an expert report (unpublished data), which provided the scientific background for prioritizing relevant risk factors. The final list of selected indicators, detailed in [25] and Table S1, was refined through expert opinion involving poultry veterinarians, AMR specialists, mathematicians, data scientists with agri-environmental backgrounds, biologists, and microbiologists. The final selection was based on consensus, prioritizing variables that met the following criteria: (i) consistent association with AMR risk in the literature; (ii) availability in international databases for the study’s spatial-temporal scope; and (iii) contextual relevance to agricultural practices in the European broiler production chain.

While the source databases provided historical data (e.g., from 1961), for this analysis, we extracted a consistent modern time series for all variables to ensure temporal coherence and relevance to contemporary agricultural practices.

Data for the selected indicators were obtained from international database collections (FAOSTAT, EuroSTAT, Our World in Data). Whenever possible, we prioritized high-resolution, long-term datasets to capture both spatial and temporal variability. The integration of multiple data sources allowed for a more comprehensive analysis of the interactions between indicators. It is important to note that the model does not produce country-specific forecasts, but rather identifies generalized, system-level risk patterns that emerge from national-scale environmental and agricultural indicators.

For regional analyses, we incorporated site-specific data provided by local agricultural agencies and research institutions. This facilitated the identification of localized patterns and trends, which are often masked in global datasets. In cases where direct measurements were unavailable, proxy variables such as land use, which influences natural habitat loss, and biodiversity that potentially affect AMR indirectly were used to estimate the values of certain indicators.

#### 2.1.2. AMR Data and Processing

The analysis utilized AMR data for bacterial isolates originating from the broiler chicken matrix (*Gallus gallus domesticus*), sourced from the EFSA and Zenodo repositories [21]. The dataset included quantitative Minimum Inhibitory Concentration (MIC) data for key pathogens and commensal bacteria, such as *Escherichia coli* and *Campylobacter*, against a range of critically important antimicrobials, including Ciprofloxacin. The data structure provided MIC ranges for each isolate–substance combination, defined by ‘lowest’ and ‘highest’ values (e.g., 0.03–16 mg/L). A critical step involved dichotomizing these continuous MIC values into resistant and non-resistant categories, which inspired the name ‘Resistant’ used in the Bayesian Network analysis. The epidemiological cut-off (ECOFF) values applied were those established for AMR monitoring in the EU, as defined by the European Committee on Antimicrobial Susceptibility Testing (EUCAST) and mandated for reporting by the European Food Safety Authority (EFSA) [21]. During the data collection period, the official EUCAST ECOFFs for certain antimicrobial–substance combinations were updated. To prevent the introduction of artifacts or pseudo-trends, all MIC data, both pre- and post-update, were retrospectively reclassified using the final, updated ECOFFs as specified in the relevant EFSA reporting requirements prior to any statistical modeling. This ensured a consistent and accurate resistance profile for all broiler-derived isolates across the entire study period.

While the environmental indicators were available in long time series, the AMR data from the EFSA repository were only available for a more recent and narrower period (2018–2023). Therefore, the temporal scope of this analysis was defined by the availability of the AMR data, and environmental indicators were extracted for this corresponding six-year period. The AMR data contained multiple bacterial isolates (a “many” relationship) for each country-year. The environmental data contained a single record (a “one” relationship) for each country-year. To preserve the full resolution of the AMR data, we performed a one-to-many merge (or “left join”). This assigned the corresponding country-year environmental–values to every individual bacterial isolate in the AMR dataset. This resulted in a final dataset where each row represents a single bacterial isolate, enriched with the environmental context of its country and year of origin. The final integrated dataset was built around the AMR isolates from the period 2018 to 2023. It included 6487 individual bacterial isolates from 20 unique countries. This is a more statistically powerful approach as it uses the full dataset and can account for within-country-year variation. The environmental indicators and AMR data used in this analysis, along with their data sources and key characteristics, are summarized in Table S1.

#### 2.1.3. Feature Engineering and Data Preparation

To create a unified dataset, we performed data merging using systematic data wrangling techniques. Following the data merging process, we addressed the issue of missing values, which is a common challenge when working with large, heterogeneous datasets. Missing data can arise for various reasons, such as incomplete reporting, data collection errors, or differences in coverage across databases. To handle this, we employed Iterative Imputation (also known as Multivariate Imputation by Chained Equations or MICE) data imputation techniques to minimize the impact of missing values on the quality and reliability of the dataset. MICE is a robust, flexible technique for imputing missing data by modeling each variable with missing values as a function of other variables, iteratively refining the imputations. It is widely supported in statistical software like R (mice package, version 3.16.0) [39] and Python 3.13.0 (Python Software Foundation, Wilmington, DE, USA).

Since the models we will use to quantify an object’s belonging to one of the classes are based on probability distributions, we need to approximate the probability distributions from the dataset. Both of the probabilistic graphical models we use are developed for discrete data. Therefore, firstly, we have to discretize the data. To do this in the most general and automatic way, we used uniform discretization (Computer Program S1), i.e., each interval contains the same number of sample data. We remark here that the discretization intervals may be undertaken in a customized way, taking into account the expert’s opinion.

The entire data pre-processing pipeline, including wrangling, merging, and imputation, was implemented in Python. We utilized pandas for data manipulation tasks and the scikit-learn library (v1.2+) for imputation [40].

#### 2.1.4. Variable Selection for BN 

The selection of BN variables was guided by a dual approach: preliminary statistical analyses to identify data-driven relationships, and the integration of domain expertise to ensure agricultural relevance. Within this framework, maize yield was explicitly included as a proxy for broiler feed supply, a critical factor in antimicrobial use patterns, and was selected for its strong performance as an environmental indicator and its alignment with EU agricultural reporting standards (geotags). This ensures the model is both statistically sound and contextually valid for analyzing the European agricultural landscape.

### 2.2. A Multi-Method Analytical Approach: From Exploratory Machine Learning to Probabilistic Graphical Models for AMR Profiling

A new complex methodology is introduced and applied based on probabilistic graphical models. This family comprises two main methods: one is related to Markov Networks, the other is related to BN. Both methods exploit in their specific way the concept of conditional independence, which will be encoded in graphs. Markov Networks use undirected graphs for the representation of multivariate random vectors, while BN uses directed acyclic graphs.

Starting from a dataset, finding the best fitting Markov Network or the best fitting BN structure is a very complex task. Therefore, we have to reduce the search space [41].

In the following subsections, we introduce the structures we used and present how this methodology was adapted for this research. We start from a preliminary step, based on which we decide which datasets contain enough information for our further graphical model-based research.

#### 2.2.1. Exploratory Classification (Machine Learning)

As a preliminary step, we assessed the feasibility of predicting AMR profiles from environmental and agricultural indicators using supervised machine learning. The dataset included resistance outcomes for multiple antibiotics—such as ciprofloxacin, erythromycin, gentamicin, tetracycline, and ertapenem—alongside explanatory variables covering zoonotic incidence, fertilizer and pesticide application, freshwater availability, climate, and demographic factors. These diverse predictors provided a broad perspective on potential ecological and anthropogenic drivers of AMR.

A key challenge was the high dimensionality of explanatory variables relative to sample size, which limited the use of complex algorithms like deep neural networks. Additionally, class imbalance favored non-resistant isolates, prompting the application of the Synthetic Minority Over-sampling Technique (SMOTE) to balance the dataset. We evaluated two machine learning algorithms: XGBoost, effective for structured data, and Kernel Support Vector Classification (KSVC), which identifies class-separating hyperplanes. This dual approach enabled performance comparison and assessment of predictive consistency across antibiotics.

Results revealed significant variation in predictive performance. Ciprofloxacin models achieved the highest performance, with XGBoost yielding an F1-score of 0.84 and an AUC of 0.79, indicating a strong predictive signal. In contrast, models for erythromycin and gentamicin were weak (F1-score < 0.20). This poor performance is primarily due to a severe class imbalance in the data for these antibiotics, characterized by an excessive proportion of non-resistant (susceptible) cases, which limited the models’ ability to learn a robust predictive pattern for resistance. These findings underscore that the association between environmental indicators and AMR is antibiotic-specific, with only certain antibiotics exhibiting predictable relationships.

These exploratory analyses served as a feasibility screen and are distinct from our primary BN analysis. While machine learning models prioritize predictive accuracy, Bayesian methods emphasize explainability and probabilistic reasoning. Our choice of BN and Generalized Naive Bayes was guided by their transparency, probabilistic output, and ability to model conditional dependencies—offering insights into system dynamics beyond predictive performance. Thus, the Bayesian framework proved more aligned with our goal of exploring environmental drivers of AMR.

#### 2.2.2. Generalized Naive Bayes Approach

**Figure 1:**
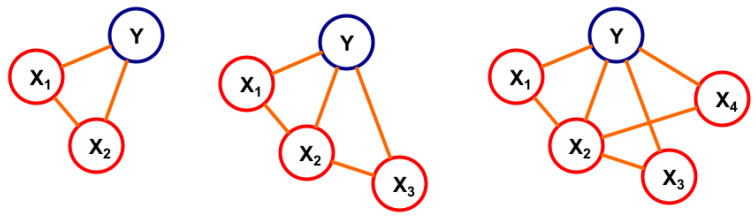
The graph illustrates the steps of the algorithm, which results in a Generalized Naive Bayes structure on a case of four explanatory variables. The process begins with the classifier variable Y (e.g., ‘Resistant’). At each step, the most informative feature is added by forming a triplet with an existing variable and Y, thereby expanding the network while minimizing redundancy. The final structure illustrates the conditional dependencies between the explanatory variables X_1_, X_2_, X_3_, X_4_ and the classifier Y, relaxing the strong conditional independence assumption of the standard Naive Bayes model.

In the class of Markov networks, the Generalized Naive Bayes (GNB) is a recently introduced approach [42]. The reasons for its introduction were the following. Naive Bayes is a very simple and useful tool, but its simplicity comes from the assumption of conditional independence between the explanatory variables given the classifier. Generally, this is not true. In the new approach, this assumption between the explanatory variables was relaxed, i.e., certain dependencies were allowed, although the structure is retained simple in order to have a reduced search space. This structure also exploits the conditional independence existing in the data, which results in using lower-dimensional marginal probability distributions. These can be derived from data of a given size in a more reliable way than the higher-dimensional ones. Another important advantage of using this model is its explainability. This comes from its graphical representation, see Figure 1.

GNB also provides probabilities for a new object belonging to each of the classes within the classifier’s range. Moreover, the construction of the GNB is enhanced with the following property: in each step of the algorithm (see Figure 1), the most informative new feature is added, i.e., it has the property of maximizing the added information taking into account all the information already in the model up to the present step. This way, redundancy is minimized at the same time. This is how this approach provides a new method for the feature selection step-by-step. The objective function to be minimized is the Kullback–Leibler divergence between the fitted probabilistic model and the real data.

The goodness of the GNB classifier is reflected by the values of the accuracy measures on the validation data. The output graph of the method shows the values of the accuracy measure of the model, after each step of the algorithm, as a function of the number of triplets used. Based on these graphs, the experts can decide how many and which explanatory features they should use.

The formula of probability assigned to the structure represented in Figure 1 is the following:

(1) $$P\left(X_{1},X_{2},X_{3},X_{4},Y\right)=\frac{P\left(X_{1},X_{2},Y\right)P\left(X_{2},X_{3},Y\right)P\left(X_{2},X_{4},Y\right)}{{\left[P\left(X_{2},Y\right)\right]}^{2}}$$

#### 2.2.3. Bayesian Network (BN)

The BN is a widely used probabilistic graphical model in probabilistic reasoning. The structure is represented by a directed acyclic graph, which exploits conditional independencies existing in the data. For this framework, packages were developed in different environments. The construction of a BN is based on a sequence of conditional independence tests. The BN model can be used in multiple ways. In the approach presented in this paper, the input consists of a dataset and the specification of the target variable and the explanatory variables. The output will be a directed graph that encodes conditional independencies, based on which certain marginal probability distributions are calculated. These are then used to calculate the probabilities for a given object to belong in each of the classes defined by the classifier. In our case, this will be resistant or not resistant to ciprofloxacin.

The general formula for the probability of a realization in a BN environment is the following:

(2) $$\prod_{i=1}^{d}P\left(X_{i}|Pa\left(X_{i}\right)\right),$$

where $X_{i}$ are the random variables (features) involved and $Pa\left(X_{i}\right)$ denotes the set of parents of $X_{i}$ in directed acyclic graph context this is $Pa\left(X_{i}\right)\to X_{i}$. If $Pa\left(X_{i}\right)=\phi$ then we have an unconditional probability of $X_{i}$.

This probabilistic setup gives the expert the possibility to find causation and to assess also probabilities for different scenarios, which are outcomes of the multivariate random vector.

#### 2.2.4. Analysis of Multi-Step Network Pathways

To quantify the influence of distant variables on the target node (Resistant), we employed a probabilistic inference approach that models the cascading effects through multiple network pathways. The baseline probability of 64% for AMR (P(Resistant = 1) = 0.640) served as the essential reference point for this analysis. This baseline is not a simple average but is algorithmically determined through probabilistic inference over the entire Bayesian network, representing the marginal probability of resistance computed by integrating across all possible states of the network’s other variables. Against this benchmark, we demonstrated how interventions on upstream variables propagate through the network architecture to influence AMR probability. For each selected multi-step route (e.g., Land_Use → Fertilizer → Maize → Resistant), we systematically altered the value of the starting variable and allowed the network to compute the cascading effect. This process was repeated under different precipitation scenarios to assess contextual modulation. The final impact was quantified by measuring the resulting probability of AMR and its net change from this baseline, revealing the degree to which the initial effect was amplified or reduced through the chain.

The intervention effects were quantified using the net change formula: Net Change = ((Final Probability-Baseline Probability)/Baseline Probability) × 100%. This metric precisely captured how initial interventions amplified or diminished through network propagation, with positive values indicating increased resistance risk and negative values showing effective risk reduction [43].

Analyses used the pgmpy Python package (version 3.13.0, 1.0.0) with custom extensions for pathway-specific inference and environmental context stratification [44].

## 3. Results

### 3.1. The Results by Applying GNB

The input of the algorithm is discretized data. First, we run the algorithm on all the explanatory variables. The output of the GNB algorithm is a graph, which illustrates how the different accuracy measures (precision, recall, f1, AUC-ROC) depend on the number of features (contained in the triplets) selected by the model.

**Figure 2:**
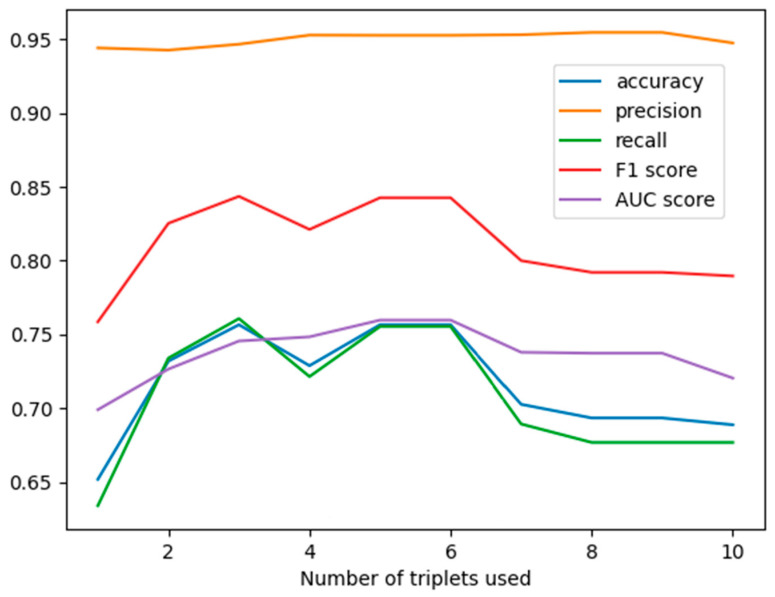
This shows the accuracy for 10 models containing one triplet, two triplets and so on. It can be observed that the model containing 6 triplets may be the best in this case.

The triplets were selected in the following order: [(‘Resistant’, ‘Pesticides’, ‘Precipitation’), (‘Resistant’, ‘Pesticides’, ‘Population’), (‘Resistant’, ‘Pesticides’, ‘Land Use’), (‘Resistant’, ‘Land Use’, ‘Fertilizer’), (‘Resistant’, ‘Population’, ‘Renewable Freshwater’), (‘Resistant’, ‘Land Use’, ‘Ghg Emissions’), (‘Resistant’, ‘Ghg Emissions’, ‘Temperature Change’), (‘Resistant’, ‘Temperature Change’, ‘Rapeseed’), (‘Resistant’, ‘Rapeseed’, ‘Soya Beans’), (‘Resistant’, ‘Soya Beans’, ‘Maize’)]. Each of the triplets contains a ‘Resistant’ classifier. The first triplet contains two explanatory features, and then each new triplet added contains one new feature. The accuracy graph on the test data is shown in Figure 2.

This graph suggests that the model using only the first six triples should be used. Based on this model we obtain accuracy: 0.7565; precision: 0.9527; recall: 0.7554; f1 score: 0.8426; AUC score: 0.7579

**Figure 3:**
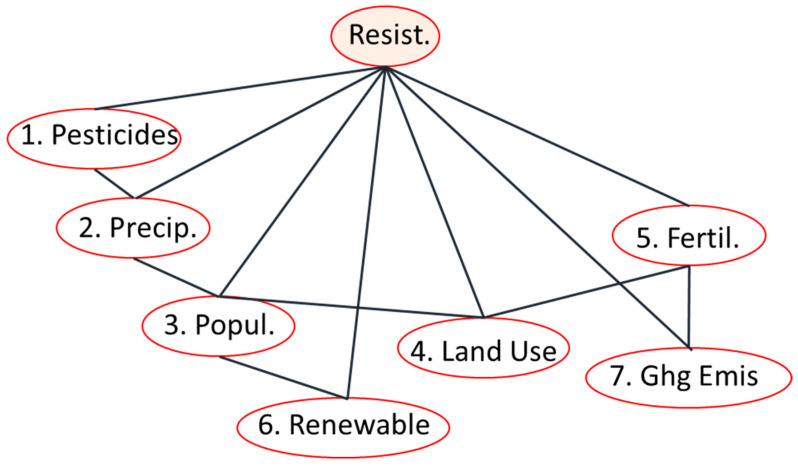
The graph of the reduced Generalized Naive Bayes (GNB) model after feature selection. The features are numbered according to their order of connection by the algorithm, with the most informative features integrated first. The structure illustrates the conditional dependencies between the seven selected explanatory variables and the target classifier, Resistant (Y). This model utilizes only second and third-order marginal probability distributions, which can be reliably estimated from the training data, to perform classification.

We highlight here that this new probabilistic graphical model uses only second- and third-order marginal probability distributions for classification, which can be reliably approximated from the training data. The graph of the reduced model can be seen in Figure 3.

Equation (3), where *Y* corresponds to Resistant (Yes or No), shows how joint probability distribution can be expressed as the product and division of third-order and second-order marginal probability distributions, each of them calculated based on the data. In this case, the explanatory variables are not forced to be independent from each other, given the classifier, like in the Naive Bayes case. In this way, by adding a new triplet, the information already in the system is taken into account, such that redundancy is minimized at each step.

(3) $$P\left(x,Y\right)=\frac{P\left(X_{1},X_{2},Y\right)P\left(X_{2},X_{3},Y\right)P\left(X_{3},X_{4},Y\right)P\left(X_{4},X_{5},Y\right)P\left(X_{3},X_{6},Y\right)P\left(X_{5},X_{7},Y\right)}{P\left(X_{2},Y\right){\left[P\left(X_{3},Y\right)\right]}^{2}P\left(X_{4},Y\right)P\left(X_{5},Y\right)P\left(X_{7},Y\right)}$$

Equation (3) gives the user the possibility to decide whether, for a given vector **x** determined by the environment, Resistant will be 1 or 0. In fact, we have to calculate the conditional probability p(Resistant|X), which is given as

(4) $$P\left(Resistant\left|x\right|\right)=\frac{P\left(x_{1},\ldots ,x_{7},Resist\right)}{P\left(x_{1},\ldots ,x_{7}\right)}$$

where the values $x_{1},..,x_{7}$ are given. Therefore, this conditional probability depends on the numerator. The Resistant class is chosen by the following formula:

(5) $$Resistant=\arg\max P\left(x_{1},\ldots ,x_{7},\;Resistant\right)$$

The argmax is taken across the two possible Resistant classes, labeled as 0 (yes) and 1 (no). In this research, we used the method for feature selection and classification based on the chosen explanatory variables. It should be highlighted that the features identified by the algorithm are not the most influential on Resistant itself, but the ones that maximize the information gained about the Resistant class.

### 3.2. Results of Bayesian Network Analysis

#### 3.2.1. Structure Learning and Robustness of the Bayesian Network Analysis

The structure of the network was learned from the data using the Hill Climbing algorithm. The optimal network structure, which achieved the best Bayesian Information Criterion (BIC) score of −41,791.81. The model reveals a complex web of interactions between crop variables, agricultural inputs, and environmental factors, culminating in the target variable ‘Resistant’.

To assess the robustness and reliability of the learned structure, a bootstrap validation (*n* = 100) was performed. This analysis quantified the arc strength, representing the proportion of bootstrap samples in which each directed relationship appears. The high stability of key arcs, particularly those directly influencing the variable ‘Resistant’, confirms that the model structure is not an artifact of random variation in the data but represents consistent probabilistic dependencies.

**Figure 4:**
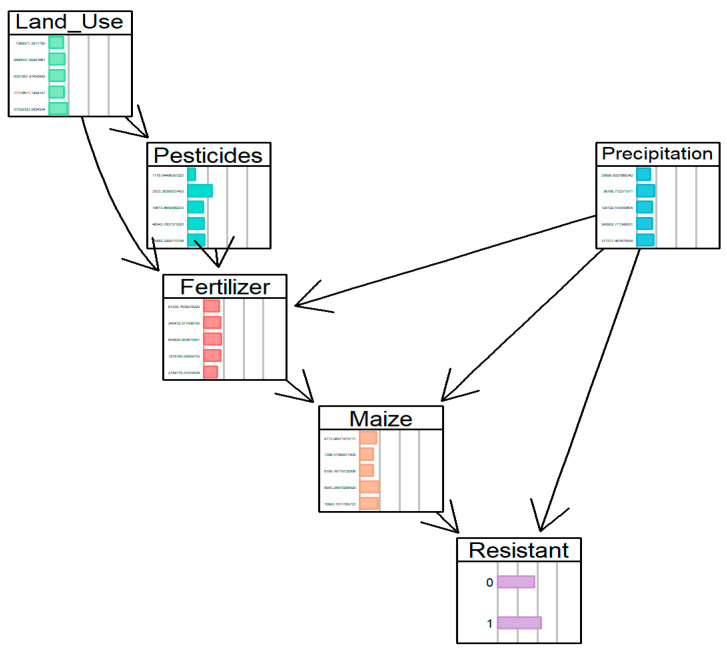
Structure of the learned BN. The directed acyclic graph depicts the probabilistic dependencies between the target variable ‘Resistant’ and the selected agri-environmental indicators. Node colors represent variable types (e.g., target, predictor), and the direction of the arcs indicates inferred conditional relationships. The network structure was learned from the discretized dataset using the Hill-Climbing algorithm and demonstrates the complex web of interactions influencing AMR probability.

The probability distribution corresponding to Figure 4 is the following:

(6) $$P\left(X\right)=P\left({Land}_{use}\right)P\left(Precip\right)P\left(Pest|{Land}_{use}\right)P\left(Fert|Land\text{\_}use,\;Pest,Precip\right)\;\;P\left(Maize|Fert,Precip\right)P\left(Resist|Maize,Precip\right)$$

#### 3.2.2. Conditional Probability Distributions (CPDs) and Predictive Insights

The BN model identifies several environmental and agricultural scenarios associated with a high predicted probability of AMR. To provide a focused analysis, we pinpointed the most significant direct factors by examining the top five scenarios for a “Resistant” outcome, based on the highest conditional probability distribution (CPD) values derived from its direct parent nodes (Maize Yield and Precipitation) within the model’s structure (Table 1).

The scenarios reveal a strong, non-linear relationship where both extremes of precipitation are associated with high AMR risk. The highest probability (*p* = 0.925) occurs under conditions of low precipitation and moderate–high maize yield (Scenario 1). Furthermore, high precipitation is a key driver in three of the top five scenarios (Scenarios 2, 3, and 5), regardless of whether the accompanying maize yield is high or low. A medium yield under low precipitation (Scenario 4) also presents a significant AMR probability.

**Figure 5:**
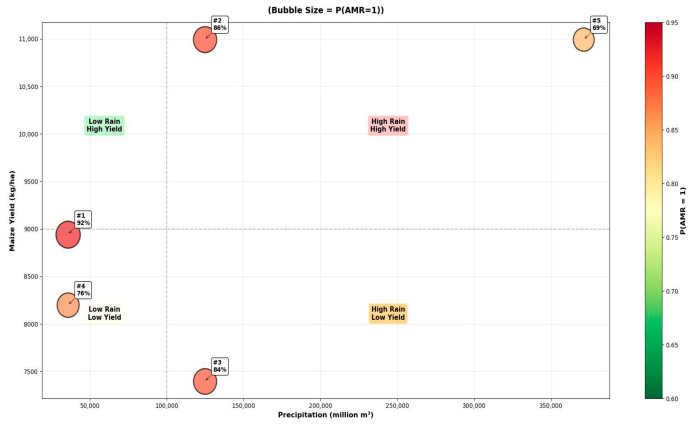
The bubble plot shows the relationship between the two direct parent nodes—Precipitation on the x-axis and Maize Yield on the y-axis—with the size and color of each bubble representing the associated probability of resistance (P(Resistant = 1)). This visualization clearly illustrates the clustering of the highest-risk scenarios (largest, reddest bubbles) at both low and high precipitation levels.

The chart demonstrates that both low and high precipitation levels are associated with a high probability of AMR. It illustrates that the scenario with very high precipitation and high yield has a lower probability than scenarios with low precipitation (Figure 5).

#### 3.2.3. Network-Wide Associations with AMR: Complex Pathways and Environmental Context

**Figure 6:**
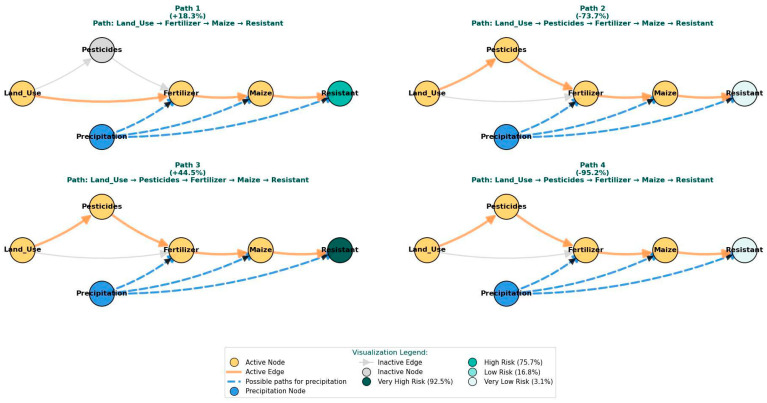
Causal pathways linking land use, agricultural activities, and precipitation to AMR. The graph highlights four main paths through the Bayesian Network, showing how changes in land use and pesticide use propagate via fertilizer and maize yield to influence resistance probability. The system’s baseline resistance probability (*p* = 0.640) is shown for reference. For each pathway, the final probability, net change from baseline, and hypothesized precipitation-mediated effects are quantified. Active nodes and edges indicate the strongest contextual influences, demonstrating how precipitation can reverse the impact of agricultural drivers.

The BN analysis indicates that the probability of AMR is associated with a complex web of factors, extending beyond direct parental influences to include multi-step pathways. In this network, environmental factors, particularly precipitation, appear to serve as critical contextual mediators that are associated with the amplification or reversal of expected outcomes. As demonstrated in Table 2 and Figure 6, variations in seemingly distant variables, such as land use, are associated with cascading effects through the network, with precipitation patterns showing a strong relationship with the ultimate direction and magnitude of the association with the target variable.

A key finding is the context-dependent association observed when changes are modeled through multiple nodes. For instance, an increase in Land_Use under conditions of low precipitation (Path 1: Land_Use → Fertilizer → Maize → Resistant) is associated with an increase in Resistant probability (+18.3%), a pattern consistent with the hypothesis of increased selection pressure in water-stressed environments [45]. Conversely, a similar Land_Use increase under high precipitation conditions (Path 2) is associated with a substantial decrease in Resistant probability (−73.7%).

The analysis also identifies what appear to be critical junctures within these pathways that are sensitive to environmental context. For example, the modeled transition to higher Pesticide use in Path 3 is associated with a sharp increase in AMR risk, but this relationship is strongly context-dependent. The most extreme case (Path 4) models a scenario where a sequence of changes under very high precipitation is associated with a −95.2% reduction in AMR probability, highlighting the potential for environmental conditions to be linked with significantly different risk profiles.

## 4. Discussion

This study demonstrates that the probability of ciprofloxacin resistance in *Campylobacter* is not a straightforward outcome of any single factor but emerges from a complex, context-dependent web of interactions within the agricultural environment. By employing a BN and a GNB model, we moved beyond simple correlative analyses to map the probabilistic relationships and multi-step pathways through which high-level environmental indicators influence resistance risk.

By building on previous results from high-level indicator analysis in broiler production, our approach contributes to early-warning capabilities and may support targeted antimicrobial stewardship in poultry systems. In the first part of this research pipeline, we developed a data-driven early-warning framework using anomaly detection (Isolation Forest) to identify atypical environmental profiles without prior knowledge of AMR outcomes. That analysis identified pesticide use, fertilizer application, land use change, and population density as dominant anomaly drivers, while precipitation and crop production (e.g., maize) emerged as context-dependent contributors. While the developed anomaly detection framework provided valuable exploratory insights by identifying atypical environmental profiles, it could not quantify the probabilistic influence of these indicators on observed resistance outcomes. The present study addresses this gap by applying BN modeling to explicitly capture the conditional dependencies between environmental variables and AMR. This approach enables the identification of the most relevant parent–child relationships, quantification of resistance probabilities under varying environmental conditions, and validation of the robustness of these relationships through sensitivity and edge-removal analyses. Together, the two studies establish a complementary analytical framework for AMR risk assessment, where anomaly detection signals unusual environmental risk profiles and BNs confirm and quantify the potential causal pathways underlying resistance emergence.

It is important to clarify the scope of our predictive model: it is designed not as a temporal forecasting tool, but as a conditional risk assessment framework. By inputting current or projected indicator values (e.g., a seasonal precipitation forecast and data on pesticide application), stakeholders can use the model to assess the concomitant risk of ciprofloxacin resistance and implement context-specific mitigation strategies before resistance manifests in the production chain.

A fundamental insight from our exploratory analysis is that the power of high-level environmental indicators to predict AMR is not universal but highly contingent on the specific antibiotic. The variability in ML model performance itself constitutes a major finding, revealing the antibiotic-specificity of environmental AMR drivers. The high performance of ciprofloxacin justified its selection for in-depth Bayesian analysis. Conversely, the weak performance for erythromycin and gentamicin stems from a severe class imbalance in the resistance data and suggests that the drivers of resistance for these classes are less dependent on the broad environmental pressures measured here. This could be due to differences in antibiotic usage patterns, environmental persistence, or the primary mechanisms through which resistance emerges. Therefore, our model should be interpreted as a successful proof-of-concept for antibiotics where resistance is strongly influenced by external environmental selection pressures; successful application of this framework to other antibiotics would require a similarly strong environmental signal and a congruent biological context.

The GNB model effectively served as a feature selection tool, identifying pesticides, land use, and precipitation as the most informative features for predicting AMR, achieving a robust F1-score of 0.8426. This initial finding signaled that agricultural intensity and climatic conditions are paramount. However, it was the subsequent BN analysis that revealed the nuanced, non-linear nature of these relationships. The BN structure, validated for robustness through bootstrap analysis, illustrated that these key indicators do not operate in a vacuum; instead, their influence is mediated through a network of conditional dependencies.

The BN analysis identified precipitation as a master environmental variable that critically modulates AMR risk pathways. The findings that both extremely low and high precipitation levels are associated with high resistance probability point to divergent underlying biological mechanisms.

Under low precipitation (drought) conditions, the risk pathway (e.g., Land_Use → Fertilizer → Maize → Resistant) likely operates through concentration effects and habitat compression. Water stress can reduce hydrological dilution, concentrating antibiotics, heavy metals, and pesticides in soil and water bodies [46,47], thereby intensifying selective pressure for resistance. Furthermore, diminished water availability can lead to increased wildlife contact with poultry operations as animals seek out limited water sources, potentially introducing or disseminating resistant strains [48].

Simultaneously, land use intensity in these dry conditions may be linked to poorer manure management outcomes; dry, compacted soils are less effective at filtering pathogens, and dust from manure-amended soils can become a vector for airborne transmission of resistant bacteria.

Conversely, under high precipitation conditions, the dominant mechanism shifts to hydrological dissemination. Heavy rainfall and surface runoff can transport resistant bacteria, antibiotic residues, and nutrients that foster their growth from agricultural lands into waterways, widely dispersing resistance determinants across the environment [34]. This runoff-mediated pathway explains the high AMR risk associated with high pesticide use and high maize yield in wet scenarios (Path 3, Scenario 2), as excess water mobilizes these agricultural inputs. Intensive land use often reduces natural vegetation buffers, exacerbating this runoff. The expansion of maize cultivation for feed, a proxy for agricultural intensity, can increase the volume of manure applied to fields, which, during heavy rain, becomes a significant pollution load, facilitating the environmental spread of AMR from livestock production systems.

The analysis of multi-step network pathways further crystallized this context-dependency. The dramatic reversal in the effect of Land_Use—from increasing AMR probability (+18.3%) under low precipitation to drastically decreasing it (−73.7%) under high precipitation along the Land_Use → Fertilizer → Maize pathway—is a powerful illustration that the same agricultural activity can have opposing consequences for AMR risk based on the environmental context. These findings challenge deterministic views of agricultural drivers and emphasize that risk is not an intrinsic property of a practice like land use intensification, but rather an emergent property of its interaction with the broader environmental system.

Furthermore, the pathway analysis suggests environmental pathway branching, where different precipitation regimes favor distinct risk routes. In drier contexts, the pathway through Fertilizer use appears dominant, supporting the hypothesis of concentrated selection pressure that may underpin scenarios like Scenario 4 (Low Precipitation, Medium Yield) [47]. This scenario may reflect a baseline where standard agricultural practices consistently contribute to selection pressure. In wetter conditions, the pathway through Pesticides gains influence, consistent with the mechanism of runoff-mediated dissemination seen in Scenario 5 (High Precipitation, High Yield), which represents a high-risk combination of agricultural activity and conditions that maximize environmental spread. This branching explains why simplistic, one-size-fits-all interventions are likely to fail; a measure that reduces risk in a dry year might inadvertently exacerbate it in a wet year.

These results support the view that AMR probability is an emergent property of the entire agricultural-environmental system as represented in our model. In this framework, precipitation appears to be a master variable that modulates the flow of association through the network. Relying solely on the direct parents of the target variable ‘Resistant’ would fail to capture these system-wide associations and the crucial contextual role of environmental conditions. The BN approach thus provides a framework for generating hypotheses about targeted interventions, suggesting that effective AMR management strategies may need to consider the complete system architecture and environmental context rather than isolated factors.

The practical utility of our BN model is a regional-level surveillance support tool. Operating at the regional scale, it allows health and agricultural authorities (e.g., within the EU) to identify member states or regions with high-risk environmental profiles. For instance, a country experiencing a forecasted dry season combined with intensive maize cultivation could be flagged for enhanced AMR monitoring in its broiler sector. The model’s output is not a farm-level prescription but a strategic guide for prioritizing surveillance resources and developing broad, context-specific advisory notices about agricultural practices. The pathways identified, such as the critical role of precipitation, provide a scientific basis for these advisories, even if on-the-ground implementation requires local adaptation.

Our approach addresses a significant gap in current AMR forecasting, which often neglects the structured complexity of agricultural systems. While traditional surveillance is retrospective, our BN framework provides a forward-looking, hypothesis-generating tool. It allows for the anticipation of resistance risk based on shifts in measurable environmental indicators, such as seasonal precipitation forecasts and data on agricultural land use. This aligns perfectly with the One Health perspective, recognizing that AMR dynamics in a foodborne pathogen like *Campylobacter* are intrinsically linked to environmental and agricultural pressures [22,23]. Our findings also underscore the critical limitation of models that consider drivers in isolation and highlight the power of a systems-thinking approach to AMR forecasting.

### 4.1. Limitations and Future Research

It is important to note that the interpretations offered above, such as the mechanisms of concentration versus dissemination, are plausible hypotheses generated by the model that require further validation rather than established causal pathways. The use of high-level proxies, while necessary for broad-scale modeling, means we are inferring underlying biological and ecological processes. Variables like maize yield serve as indicators of intensive agricultural activity but do not capture on-farm antibiotic use practices. Future work would benefit from integrating more direct, granular data on antimicrobial usage in livestock, as well as molecular data on resistance genes and mobile genetic elements, to validate the hypothesized pathways. Additionally, the model’s performance is contingent on the quality and resolution of the international databases used. Expanding the dataset temporally and geographically will be crucial for testing the generalizability of the identified networks.

A key methodological consideration is that the GNB graph and the associated probability distribution are dependent on the discretization used. If a different discretization is required, the algorithm must be run again. Furthermore, both probabilistic graphical model-based methodologies use discrete data as input; this way, we do not use all the information contained in the continuous data. As future work, we plan to extend the GNB method to also deal with continuous explanatory variables to enhance the model’s precision and leverage the full information content of the data.

### 4.2. Towards a Proactive Early-Warning System for AMR Surveillance

The findings of this study, when integrated with our previous work on environmental anomaly detection [25], lay the groundwork for a transformative, two-tiered early-warning system for AMR. This integrated framework moves beyond retrospective surveillance towards a proactive, data-driven approach for managing AMR risk in the broiler production chain.

**Figure 7:**
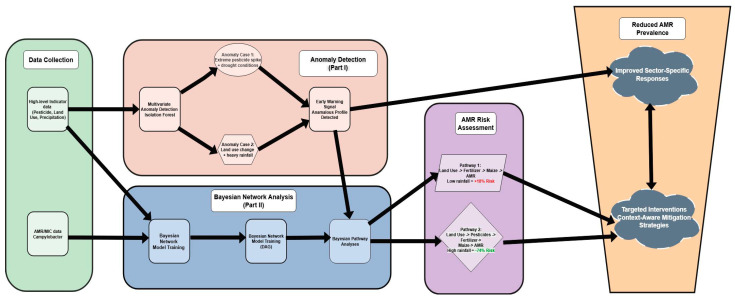
Schematic of the integrated two-stage early-warning system for AMR. The framework combines unsupervised anomaly detection of environmental indicators (Stage 1) with a Bayesian network for probabilistic risk assessment and pathway analysis (Stage 2). This integration enables both rapid, general alerts for multiple sectors and targeted, context-aware interventions for the poultry production chain.

The first stage of this system, as demonstrated in our prior research, employs unsupervised anomaly detection (Isolation Forest) to continuously monitor high-level environmental indicators. This layer acts as a sensitive and rapid “smoke alarm,” flagging unusual environmental profiles—such as a combination of extreme pesticide use and drought conditions, or significant land use change during periods of heavy rainfall—without the need for prior AMR data. This provides a crucial general alert for public health, agricultural, and environmental agencies, enabling sector-wide vigilance (Figure 7).

The second stage, detailed in the present study, utilizes BN analysis to provide a deep, causal diagnosis of the alerted anomalies. When an unusual environmental profile is detected, the BN model interprets it through probabilistic pathways. For instance, our analysis reveals that an anomaly characterized by increased land use elevates AMR risk by 18.3% under low precipitation (via the Land Use → Fertilizer → Maize → AMR pathway), yet it decreases risk by 73.7% under high precipitation. This pathway-specific intelligence transforms a generic alert into a context-aware early warning. It answers not just *that* there is a risk, but *why* the risk exists and *how* it is modulated by environmental conditions.

In practice, this means that a forecast of a dry season could trigger an early warning to limit fertilizer application, mitigating the risk of concentrated selection pressure. Conversely, a forecast of a wet season would generate a warning to control pesticide runoff, thereby reducing the risk of widespread environmental dissemination. By marrying the speed and breadth of anomaly detection with the explanatory depth and predictive power of Bayesian networks, this integrated system enables targeted, pre-emptive interventions.

Ultimately, this research provides a scalable blueprint for shifting the paradigm in AMR management from reactive to proactive. Future work will focus on implementing this early-warning system in real-time, using live data streams to validate its efficacy in guiding antimicrobial stewardship and protecting public health within a true One Health framework.

## 5. Conclusions

In conclusion, our graph-based machine learning and Bayesian approach successfully demonstrates that ciprofloxacin resistance in *Campylobacter* is an emergent property of a complex system.

In the analytical framework, the risk of resistance was not determined by agricultural indicators alone but by their interaction with key environmental predictors, most notably precipitation. This explains why the same agricultural activity can lead to vastly different resistance outcomes.

AMR probability arose from cascading effects through multi-step pathways within the network. Focusing solely on direct, proximal factors to AMR provides an incomplete and potentially misleading picture.

Therefore, effective antimicrobial stewardship in the poultry production chain must extend beyond the farm gate to include landscape-level environmental monitoring. Our BN approach provides a valuable framework for developing early-warning systems that can anticipate fluctuations in AMR risk based on forecasts of environmental conditions, enabling more targeted, pre-emptive, and context-aware interventions to mitigate the spread of AMR.

These findings, combined with our previous work on environmental anomaly detection [25], pave the way for a two-tiered proactive early-warning system. The first tier employs anomaly detection as a rapid “smoke alarm” to flag unusual environmental profiles, providing crucial alerts for sector-wide vigilance. The second tier utilizes the BN for a deep, causal diagnosis, interpreting these anomalies through probabilistic pathways to deliver context-aware early warnings. By connecting the speed of anomaly detection with the explanatory power of BNs, this integrated system can transform AMR management from a reactive to a proactive discipline. Future work will focus on implementing this system with real-time data streams, validating its power to guide targeted interventions and protect public health within a robust One Health framework.

## Figures and Tables

**Table 1:** Top 5 scenarios for high resistance probability. This table presents the five most probable scenarios for ciprofloxacin resistance (Resistant = 1) as determined by the BN model, conditioned on its direct parent nodes: Maize and Precipitation.

Rank	P (Resistant = 1)	Maize Yield (kg/ha)	Precipitation (Million m^3^)	Interpreted AMR Pathway
1	0.925	Moderate–High (8943)	Low (35,698)	Selection Pressure in Water-Stressed Environments
2	0.856	High (10,993)	High (124,722)	Runoff-Mediated Dissemination
3	0.840	Low (7396)	High (124,722)	Environmental Spread Independent of Yield
4	0.757	Medium (8199)	Low (35,698)	Baseline Agricultural Selection Pressure
5	0.687	High (10,993)	Very High (371,311)	Saturated System with High Dissemination

**Table 2:** Pathway analyses. This table demonstrates the cascading, context-dependent effects propagated through the Bayesian network. Together, Table 2 and Figure 6 illustrate that the risk of antimicrobial resistance is not a simple function of any single driver but an emergent property of the entire system. The baseline probability of resistance (*p* = 0.640) serves as the reference. For each pathway, an intervention increasing Land Use (in million hectares, M ha) is modeled, showing the consequent changes in downstream variables (Pesticides ‘Pest’ and Fertilizer ‘Fert’ in tons; Maize Yield in kg/ha).

Pathway	Land Use Intervention	Key Effects	Precipitation Role
Baseline	-	-	-
Path 1	1.37 M→2.85 M	Fert: 61 K→245 K Maize: 5 K→8 K	Low precipitation enables selection pressure
Path 2	2.85 M→9.36 M	Pest: 4 K→11 K Fert: 245 K→606 K Maize: 8 K→11 K	High precipitation drives runoff-mediated dilution
Path 3	9.36 M→17.11 M	Pest: 11 K→47 K Fert: 606 K→1.22 M Maize: 11 K→9 K	Moderate precipitation optimizes environmental persistence
Path 4	17.11 M→27.55 M	Pest: 47 K→66 K Fert: 1.22 M→2.18 M Maize: 9 K→7 K	Very high precipitation causes system flushing and dilution

## Data Availability

The original contributions presented in this study are included in the article. Further inquiries can be directed to the corresponding author.

## Supplementary Materials

Table S1. Selected indicators, data sources, and their characteristics: https://github.com/Csorbasz/amr_and_environmental_raw_data/blob/main/AMR%20%26%20indicators%2C%20data%20sources%20and%20their%20characteristics.pdf (accessed on 6 October 2025). Computer Program S1. The discretization: QuantileDiscretize/quantileDiscretize.py at main · DaniPfeifer/QuantileDiscretize · GitHub (https://github.com/DaniPfeifer/QuantileDiscretize/blob/main/quantileDiscretize.py) (accessed on 6 October 2025).

## Abbreviations

The following abbreviations are used in this manuscript:

AMR	Antimicrobial resistance
ARG	Antimicrobial resistance gene
AUC	Area under the curve
BIC	Bayesian information criterion
BN	Bayesian network
CFU	Colony forming unit
CPD	Conditional probability distribution
EFSA	European Food Safety Authority
ECOFF	Epidemiological cut-off
EUCAST	European Committee on Antimicrobial Susceptibility Testing
FAOSTAT	Food and Agriculture Organization Statistical Database
F1-score	F-measure (harmonic mean of precision and recall)
GNB	Generalized naive Bayes
HPC	Hill climbing (algorithm)
KL Divergence	Kullback–Leibler divergence
KSVC	Kernel support vector classification
MIC	Minimum inhibitory concentration
ML	Machine learning
PGM	Probabilistic graphical model
ROC	Receiver operating characteristic
SMOTE	Synthetic minority over-sampling technique
XGBoost	Extreme gradient boosting
